# Repeated stress exposure leads to structural synaptic instability prior to disorganization of hippocampal coding and impairments in learning

**DOI:** 10.1038/s41398-022-02107-5

**Published:** 2022-09-12

**Authors:** Alireza Chenani, Ghabiba Weston, Alessandro F. Ulivi, Tim P. Castello-Waldow, Rosa-Eva Huettl, Alon Chen, Alessio Attardo

**Affiliations:** 1 Max Planck Institute of Psychiatry, 80804 Munich, Germany; 2 Graduate School of Systemic Neurosciences GSN-LMU, 82152 Munich, Germany; 3 Leibniz Institute for Neurobiology, 39118 Magdeburg, Germany; 4 Weizmann Institute of Science, 76100 Rehovot, Israel

**Keywords:** Hippocampus, Long-term memory

## Abstract

Stress exposure impairs brain structure and function, resulting in cognitive deficits and increased risk for psychiatric disorders such as depression, schizophrenia, anxiety and post-traumatic stress disorder. In particular, stress exposure affects function and structure of hippocampal CA1 leading to impairments in episodic memory. Here, we applied longitudinal deep-brain optical imaging to investigate the link between changes in activity patterns and structural plasticity of dorsal CA1 pyramidal neurons and hippocampal-dependent learning and memory in mice exposed to stress. We found that several days of repeated stress led to a substantial increase in neuronal activity followed by disruption of the temporal structure of this activity and spatial coding. We then tracked dynamics of structural excitatory connectivity as a potential underlying cause of the changes in activity induced by repeated stress. We thus discovered that exposure to repeated stress leads to an immediate decrease in spinogenesis followed by decrease in spine stability. By comparison, acute stress led to stabilization of the spines born in temporal proximity to the stressful event. Importantly, the temporal relationship between changes in activity levels, structural connectivity and activity patterns, suggests that loss of structural connectivity mediates the transition between increased activity and impairment of temporal organization and spatial information content in dorsal CA1 upon repeated stress exposure.

## Introduction

Stress exposure impairs brain structure and function, resulting in cognitive deficits and increased risk for psychiatric disorders such as depression, schizophrenia, anxiety and post-traumatic stress disorder [1, 2]. While the mechanisms by which stress leads to the appearance of these psychiatric disorders are not completely understood, it is clear that chronic stress affects the structure and physiology of the hippocampus [3–6] and leads to long-lasting spatial memory impairments [7, 8]. In rodents, structural changes include shrinkage of dendritic trees and changes in the density of dendritic spines [9–11]. Physiological harms include decreased Long Term Potentiation (LTP) and increased Long Term Depression (LTD) [12–16] as well as dysregulated place cells firing in the CA1 region [16–20]. All these changes alter hippocampal representations in ways that are largely unknown and might underlie memory impairments. Deciphering such alterations in rodents will help establishing endophenotypes of stress-associated disorders and could potentially guide new strategies for intervention.

Importantly, the link between structural and physiological changes in the hippocampus upon stress exposure remains largely unexplored, owing mostly to technical limitations. First, the methods used to record neuronal activity have confined studies investigating changes in hippocampal activity upon stress exposure to a relatively small number of place cells. This prevented a broad understanding of the effects of repeated stress exposure on hippocampal coding. Second, the need to sacrifice the animals in order to study their neuronal structure precluded longitudinal studies of long-term structural synaptic dynamics during repeated stress exposure. These dynamics—in the neocortex—are associated with the ability to learn [21–24], play an important role in the action of antidepressants [25] and mediate the cognitive effects of stress exposure [26]. Third, most studies focused either on structure or function and applied different stress paradigms and model systems to dissect structural plasticity and activity separately. Thus, linking changes in connectivity and activity still remains difficult.

To solve these issues, we employed deep-brain optical imaging to study pyramidal neurons (PNs) located in the dorsal aspect of hippocampal CA1 (dCA1) of mice undergoing repeated stress exposure. Optical imaging gave us the opportunity to study large populations of neurons at variable temporal—from hundreds of milliseconds to weeks—and spatial—from micrometers to millimeters—scales. Importantly, optical imaging enabled us to perform long-term longitudinal studies on the same subjects, thus allowing us to normalize to baseline conditions within the same individuals. This is especially important in the hippocampus where the presence of substantial turnover of both spatial coding and structural connectivity [27–31] makes it crucial to take into account changes in structure or activity patterns due to passing of time.

We subjected mice to repeated stress and investigated the activity patterns of dCA1 PNs, by using Wide Field Head-Mounted (WFHM) optical imaging and studied the structural dynamics of dendritic spines—as proxies for excitatory synapses—, by employing two-photon (2 P) optical imaging. We finally investigated the effects of the same stress exposure paradigm on the ability of mice to learn and recall a hippocampal-dependent learning and memory task. Repeated stress exposure: (i) immediately and sustainedly increased the amount of neuronal activity in the dCA1 but decreased its temporal organization and spatial coding, (ii) led to decrease in synaptic structural connectivity which occurred in two steps—an initial decrease in spinogenesis followed by increased spine loss—and (iii) impaired the ability of mice to learn the location of the hidden platform in a Morris water maze task.

## Materials and Methods

### Subjects

Animals were either C57BL6 (for WFHM freely-moving imaging) or Thy1-GFP (M line) transgenic (for 2 P head-fixed imaging) male mice between 3 and 6 months of age. Animals had free access to food and water with a 12/12 light/dark cycle. All animal procedures conformed to the guidelines of the Max Planck Society and the local animal authority (Regierung von Oberbayern—Veterinärwesen) and were approved in the License for animal experimentation # ROB-55.2Vet-2532.Vet_02-16-48.

### Viral vector injection

Intracranial injections of Adeno Associated Viral suspensions were carried out according to standard methods. We injected 500 nL of a viral suspension (AAV2/1.Syn.GCaMP6f.WPRE.SV40; titer, 7.2 × 10^12^ genomes copy/ml; U Penn Vector Core) at a rate of 100 nL/min in dCA1 (AP, -2.4 mm; ML, 1.5; DV, 1.3 mm). Mice were allowed to recover for a minimum of 10 days.

### Implantation of a chronic hippocampal imaging window

We implanted chronic hippocampal imaging windows as previously described [30]. A metal cannula (2.5 mm-inner-diameter, 1.6 mm-long) was inserted into a 3 mm-diameter craniotomy centered at −2.4 mm, AP, 1.5 mm ML relative to bregma. The cannula was fixed and sealed to the skull using Metabond (Parkell). A custom head plate was positioned and fixed with dental cement.

For WFHM imaging, the head plate consisted of a plastic cylinder threaded to enable mounting of the microscope objective and the microscope (Doriclenses).

For 2 P imaging, the head plate consisted of a thin aluminum slab with holes to fit a holder positioned on the 2 P microscope stage.

### In vivo optical imaging

For WFHM imaging, we familiarized mice with the recording room and the circular arena for 3 days. The objective lens (0.5 NA, 2.4 WD, Air immersion) and the microscopes (Doriclenses, S model) were mounted on the head of the animals, and the animals were free to explore the arena for 5–10 min/day. On the last day of habituation, we rotated the objective lens to reach a depth at which we could detect neuronal activity. We then glued the objective in place using epoxy glue and removed the miniaturized microscope before placing the animals back in their home cage. During the 14 days following the habituation period, we mounted the miniaturized microscope to the imaging objective, placed the animals in the same arena each day and recorded Ca^2+^ transients for 15 min at a sampling rate of 45 Hz using commercial software (Doric Neuroscience Studio software) and 488 nm continuous wavelength laser (Thorlabs) illumination (1 mW average power). We cleaned the arena and changed the bedding every day and mixed oat flakes in the bedding material to motivate exploration.

For 2 P imaging, we anesthetized the mice with 2.5% isoflurane and placed them onto a heating pad (CMA 450) under the microscope (Bruker Ultima IV) while the head was fixed to a holder. Mice were kept under constant anesthesia (1.5% isoflurane). We aligned the imaging cannula to the light path as previously described [31]. We used a 25x water immersion objective (Olympus XLPlan N 25x/1.00 SVMP) and a pulsed infrared laser tuned to 920 nm. We acquired z-stacks (a square surface with each side measuring 48.18 μm, 1 μm z-step, 5-60 z-steps, 28.6-115.5 mW laser power at the sample) using a resonant scanner at each time point.

*For WFHM imaging*, we first performed data reduction using CaImAn then spatially down-sampled the image time series by a factor of 2 and motion corrected using the NoRMCorre algorithm [32]. We simultaneously denoised, deconvolved, and demixed our data using the extended version of the constrained non-negative matrix factorization (CNMF-E) algorithm [33]. This method uses sparse non-negative deconvolution [34] to estimate the beginning of the rise of the GCaMP6f fluorescence. We finally time-stamped the deconvolved signal for all neurons. The parameters used for the CNMFE algorithm are reported in Supplementary table 1.

*Activity rates* of neuronal populations were calculated as the sum of individual neuronal activity rates during the imaging session divided by the number of neurons in each session

$$\frac{{{\sum} {r_i} }}{{N_{neurons}}}$$

where *r*_*i*_ is the average activity rate for the i-th neuron. *Population rate* is the sum of all binarized neuronal activity per time bin (22 ms). *Population bursts* were defined as events in which the population rate reached at least 2.5 standard deviations (S.D.) above its mean. The *population burst duration* was the time span where population rate stayed above 1 S.D. above of its mean. *Population burst rate* was the number of population bursts recorded in each session divided by the total recording time. *Participation to population bursts* was defined as the fraction of population bursts that a neuron contributed to, normalized to the total number of population bursts. The heuristic *threshold for higher activity rate PNs* was defined as the point at which the linear relationships between activity rate and participation in bursts becomes different between the baseline and stress periods, this corresponded to the mean baseline activity rate +1 S.D. (activity rate > 1 Z-score). To compute the *power spectral density* of population activity, we collapsed all calcium events from all neurons and performed a Fast Fourier Transform (as implemented in the Spectrum software package [35]) on the rate of activity. The higher sampling rate of our system (45 Hz) bound our analysis to frequencies up to 22 Hz, according to the Nyquist theorem which states that the highest frequency that can be represented accurately is one half of the sampling rate. The spectra of control and repeated stress groups were normalized to the average spectrum of the baseline. We calculated the *pairwise Pearson correlation matrices* per mouse at each time point by using neuronal activity time series binarized and binned within 132 ms bins. We then generated *positive correlation adjacency matrices* by selecting only positive Pearson correlation values and by binarizing the correlation matrices in comparison to 95 percentile correlation values for correlations among shuffled data set of neurons with scrambled activity rate. We could thus generate co-activation networks whose nodes were the PNs and whose edges - or connectivity strengths—were the values of pairwise temporal correlation between neurons (Supplementary Fig. 4c). Network *Modularity and Assortativity* were calculated using Newman’s spectral community detection implemented in Brain Connectivity Toolbox [36]. We defined *neuronal ensembles* as the principal components of the pairwise Pearson correlation matrices [37] and their *encoding strengths* as the ratio between ensemble eigenvalue (*λ*) and the Marcenko-Pastur upper threshold *λ*_*max*_.

$$Reactivation\,Strength = \frac{{\uplambda }}{{\lambda _{max}}}$$

*Significant ensembles* were defined as ensembles with eigenvalues exceeding Marcenko-Pastur threshold [37]; only significant ensembles were considered for further analyses. We defined the *ensemble core* as the fraction of neurons with weights greater than half of maximum weight for a given ensemble. We defined *the participation index to ensembles* of a neuron as the number of significant ensembles having that neuron as a part of their core divided by total number of significant ensembles in the recording session. To obtain *spatial activity maps* we determined the location of the animal at the onset of each calcium event and we divided the arena into 5 cm × 5 cm non-overlapping bins. We then calculated the activity rate in each bin as the number of calcium events for each bin divided by the occupancy time of that bin. Only calcium events where animal speed exceeded 2 cm/s were considered in the analysis. *Mean activity rates* were calculated by averaging the activity rate of each neuron. *Spatial information* was calculated as previously described [38].

$$SI = \mathop {\sum }\limits_{i = 1}^N p_i\frac{{r_i}}{{\bar r}}\log _2\left( {\frac{{r_i}}{{\bar r}}} \right)$$

where _*pi*_ is the occupancy probability, *r*_*i*_ is the average activity rate of the i-th bin and $\overline r$ is the average firing rate of the neuron. The spatial information value was Z-scored to the distribution of spatial information values obtained by shuffling the activity times of the neuron 100 times. We used positive and negative values of Pearson’s correlations to generate *connectivity networks* whose nodes were the PNs and whose edges were defined as positive connections for positive values or as negative connections for negative values of pairwise temporal correlation between PNs. For each PN we calculated a *connectivity index* as the algebraic sum of all its connectivity, normalized by the total number of its connections.

*For 2* *P imaging*, we compensated for motion artifacts and scored as previously described [31]. Fractional gain and fractional loss between two consecutive imaging points were defined as the number of spines gained or lost between the first and the second time points, normalized by the number of spines present in the first time point. Fractional survival was defined as the number of spines surviving between the first and each of the other time points, normalized by the number of spines present in the first Baseline time point, first and second stress time points. The experimenter was blinded to the baseline or stress periods.

### Multimodal stress

Multimodal stress exposure was performed as previously described [39, 40]. Each mouse was kept in a restrainer that allowed breathing and had a slot for the headplates. All restrainers with mice of an experimental cohort were fixed together on a rocking plate. Mice were kept under loud noise and high illumination conditions for the duration of the restraint (2 h). Repeated stress consisted of seven daily 2 h-long sessions while acute stress was a single 2 h-long session.

### Analysis of animal behavior

Morris water maze. The animal behavior was recorded during navigation of a standard (circular, 2 m diameter, filled with water at a temperature of 24 degrees Celsius) water maze arena. We used commercial software (Anymaze, Stoelting) to extract the position and speed of animals from video recordings and calculated latency and quadrant occupancy.

Exploration of the open arena. The animal behavior was recorded simultaneously with neuronal recording during 15 min-long exploration of a circular arena (40 cm diameter). Data are averages per animal during a session. The position and speed of animals were extracted from a live video feed using a custom made Bonsai [41] script. Animal’s trajectories were corrected manually for transient tracking errors. Speed is length of path covered by animals per unit time, Immobility is the percentage of time during which animals showed speed = 0, Occupancy is the percentage of the surface area of the arena the animals explored, Center time is the percentage of time the animals spent in the center of the arena. The center of the arena was defined as a circle concentric with the arena and with the dimeter equal to ½ of the arena’s diameter.

### Statistical analysis

Mice of the same age- and gender matched cohort were randomly assigned to each group. We choose sample size according to previous literature. We analyzed 4 mice in the Control group and 9 mice in the Repeated stress group in the WFHM imaging experiments (See Supplementary table 2 for number of neurons per mouse per day). We analyzed 6 mice (114 dendrites tracked) in the Repeated stress and 4 mice (88 dendrites tracked) in the Acute stress group in the 2 P imaging experiments. We analyzed 10 mice each in the Control, Repeated and Acute stress groups in the water maze navigation experiments. With the only exception of SI, Z-scores were calculated on the distributions of values over the baseline period per mouse. Specifically, for each data point of control and stress periods we subtracted the mean and divided by the S.D.s of the baseline distributions per each mouse. We used Pearson’s correlations, Kruskal–Wallis tests with Dunn’s corrections for multiple comparisons, 2-way ANOVA with Tukey’s corrections for multiple comparisons, Mann–Whitney *U*-tests, Wilcoxon matched pairs signed ranks tests with Dunn’s correction for multiple comparisons, Pairwise, two-tailed *t*-tests with Holm-Šidák correction for multiple comparisons, one sample *t*-tests. Statistical analysis and plotting were done with Prism 9 (GraphPad) or Python software.

## Results

### Imaging activity of hundreds of neurons in freely-moving mice undergoing repeated exposure to stress

**Fig. 1:**
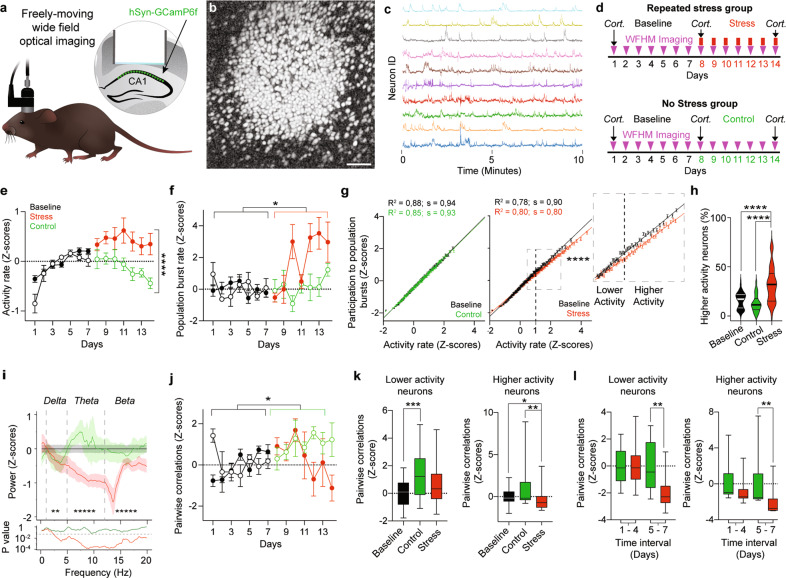
Exposure to repeated stress induces hyperactivity but reduces activity coordination in dCA1. **a** Schematic description of WFHM optical imaging of neuronal activity in the dCA1 of freely-moving mice. **b** Neurons active during a 15-min-long exploration of a round arena. Gray scale values in the image represent the correlation of that pixel brightness in one imaging frame with the same pixel brightness in all other imaging frames, for the full duration of the recording session. Scale bar, 80 µm. **c** Example raw Ca^2+^ traces extracted from a WFHM 10 min-long recording of dCA1. **d** Experimental timelines for mice stressed repeatedly (left) or not stressed (right). Cort. indicates measurements of plasma corticosterone levels. **e** Stress exposure increased neuronal activity, for statistical analysis we used baseline data of days 5, 6 and 7 to exclude artifacts due to increase in activity from days 1–4 (*p*_Bcontrol-Bstress_ > 0.99, *p*_Bcontrol-C_ = 0.51, *p*_Bstress-S_ = 0.40, *p*_C-S_ < 0.0001; *n*_Bcontrol_ = 12, *n*_Bstress_ = 49, *n*_C_ = 27, *n*_S_ = 49). **f** Stress exposure increased population burst’s rates (*p*_Bcontrol-Bstress_ > 0.99, *p*_Bcontrol-C_ > 0.99, *p*_Bstress-S_ = 0.019, *p*_C-S_ = 0.25; *n*_Bcontrol_ = 27, *n*_Bstress_ = 59, *n*_C_ = 27, *n*_S_ = 49). **g** Activity rate correlated with participation in population bursts of PNs (**g** *R*^2^_B_ = 0.88, *R*^2^_C_ = 0.85; *p*_B_ < 0.0001, *p*_C_ < 0.0001; *n*_B_ = 13862, *n*_C_ = 13057. **h** *R*^2^_B_ = 0.78, *R*^2^_S_ = 0.80; *p*_B_ < 0.0001, *p*_S_ < 0.0001; *n*_B_ = 20664, *n*_S_ = 19858). Stress exposure changed this relationship (*p*_B-S_ < 0.00001, *n*_B_ = 13862, *n*_S_ = 19858; Two-way ANOVA) due to a reduction in participation of neurons with higher activity rates (neurons with activity rate > 1 Z-score, inset). Circles: participation index of neurons in population bursts binned over activity rates. Lines: linear fit to the data. Error bars: s.e.m. **h** Stress exposure doubled the percentage of neurons with higher activity rates (*p*_B-C_ = 0.4, *p*_B-S_ < 0.0001, *p*_C-S_ < 0.0001; *n*_B_ = 87, *n*_C_ = 28, *n*_S_ = 49). Violin plots: median and quartiles of the percentage of neurons with higher activity rates distributions. **i** Stress exposure decreased neuronal synchronization in the Delta (1–5 Hz), Theta (5–12 Hz) and Alpha (12–20 Hz) bands (Delta: *p*_B-C_ < 0.00001, *p*_B-S_ < 0.00001, *p*_C-S_ = 0.0094; *n*_B_ = 1357, *n*C = 529 *n*S = 506. Theta: *p*_B-C_ = 0.0006, *p*_B-S_ < 0.00001, *p*_C-S_ < 0.00001; *n*_B_ = 2400, *n*_C_ = 920, *n*_S_ = 960. Alpha: *p*_B-C_ < 0.00001, *p*_B-S_ < 0.00001, *p*_C-S_ < 0.00001; *n*_B_ = 2700, *n*_C_ = 1035, *n*_S_ = 1080; Mann–Whitney *U*-test). Asterisks: *p*-values for the comparisons of Control and Stress. Bottom panel: *p*-values of the comparisons of Control (green) and Stress (red) powers with Baseline (bin width = 0.17 Hz) throughout the spectrum. **j** Stress exposure blocked increase in pairwise temporal correlations in all neurons (*p*_Bcontrol-Bstress_ > 0.99, *p*_Bcontrol-C_ = 0.0378, *p*_Bstress-S_ = 0.38 0., *p*_C-S_ = 0.28; *n*_Bcontrol_ = 22, *n*_Bstress_ = 53, *n*_C_ = 25, *n*_S_ = 42). **e**, **f**, **j** Circles: mean activity rates **e** population burst rates **f** or pairwise temporal correlations **j** per mouse, Z-scored over the baseline of each mouse. Solid symbols, stress group. Empty symbols, control group. Error bars: s.e.m. **k** Correlation of lower activity neurons significantly increased during the second week of imaging in the control group (left: *p*_B-C_ = 0.0008, *p*_B-S_ = 0.083, *p*_C-S_ = 0.3; *n*_B_ = 75, *n*_C_ = 25, *n*_S_ = 42). The difference between Control and Stress groups became significant after 5 days of repeated stress (right: *p*_8–11_ > 0.99, *p*_12–14_ = 0.0038; *n*_C 8–11_ = 16, *n*_S 8–11_ = 28, *n*_C 12–14_ = 9, *n*_S 12–14_ = 14). **l** Stress exposure decreased the temporal correlation of higher activity neurons (left: *p*_B-C_ = 0.36, *p*_B-S_ = 0.025, *p*_C-S_ = 0.002; *n*_B_ = 75, *n*_C_ = 24, *n*_S_ = 42). The difference between control and stress groups became significant after 5 days of repeated stress (right: *p*_8–11_ = 0.11, *p*_12–14_ = 0.0039; *n*_C 8–11_ = 15, *n*_S 8–11_ = 28, *n*_C 12–14_ = 9, *n*_S 12–14_ = 14). Data in each time interval were normalized to the mean of the control group in that interval. **e**, **f**, **h**, **j**, **k**, **l** Kruskal–Wallis test, *p-*values adjusted after Dunn’s corrections for multiple comparisons.

We used WFHM fluorescence microscopy (Fig. 1a) to study the activity patterns of hundreds of dCA1 PNs in freely-moving mice. Three to 4 weeks after viral transfection of the dorsal CA1 with Adeno Associated Viruses encoding for the Ca^2+^ sensor GCaMP6f [42], we repeatedly imaged Ca^2+^ transients in dCA1 PNs (Fig. 1b, c) in mice exploring a circular arena for 15 min every day for 7 days (Fig. 1d). We then divided the mice in two groups. The repeated stress group underwent 2 h of multimodal stress [39, 40] exposure every day for 7 days. Briefly, each mouse was kept in a restrainer– that allowed breathing and had a slot for the headplates –, all restrainers with mice of an experimental cohort were fixed together on a rocking plate. Mice were kept under loud noise and high illumination conditions for the duration of the restraint. Stress exposure ended at least 2 h prior to each imaging session. The control (No Stress) group was imaged every day for seven additional days without stress exposure (Fig. 1d). We imaged up to 1356 neurons in a single session and an average of 393 (±24 s.e.m.), 468 (±78 s.e.m.) and 405 (±50 s.e.m.) neurons per mouse per session during the baseline, control and stress periods respectively. The number of neurons did not significantly change over time or across groups (Supplementary Fig. 1a, b).

Basic parameters of exploration behavior such as mouse speed, immobility, occupancy of the arena and of its center, did not show a significant time dependency during baseline (Supplementary Fig. 2). However, repeated stress exposure decreased average speed, increased immobility and decreased occupancy of the arena (Supplementary Fig. 2a–i) but did not affect permanence in the center of the arena (Supplementary Fig. 2j–l) which is often used as a measure of anxiety.

Upon repeated stress exposure, we found high levels of circulating corticosterone. However–owing to high baseline levels–stress-induced increase in circulating corticosterone did not reach statistical significance over baseline (Supplementary Fig. 3a–c).

### Exposure to repeated stress induces PN hyperactivity in dCA1

The activity rate in the prospective Control and the Stress groups increased during the baseline period until day 4 and stabilized from day 5 onwards (Supplementary Fig. 4a). Exposure to repeated stress increased PNs’ activity (Fig. 1e) and population burst rates (Fig. 1f) but it decreased the population burst’s duration (Supplementary Fig. 4b). Repeated stress exposure reduced the participation of highly active neurons in population bursts (Fig. 1g) but it almost doubled the number of PNs with higher activity rates (Fig. 1h). Activity rates showed a very weak correlation with average speeds and center times during baseline, but these correlations were not significant during the stress period (Supplementary Fig. 2m).

### Exposure to repeated stress reduces temporal coordination in dCA1

We then investigated whether an increase in activity would lead to increased synchronization of dCA1 PNs. To this aim we examined several important measures of network synchronization. First, as the fast sampling rate of our system (45 Hz) enables us to represent accurately (or detect) frequencies up to 22 Hz, we used Fast Fourier Transform on the population activity data to extract the power at different frequencies reaching up to a good portion of Beta waves frequencies. Exposure to repeated stress significantly decreased the powers of the Delta (1–5 Hz), Theta (5–12 Hz) and Beta (12–20 Hz) frequency bands, with the largest difference between control and stress groups being in the Theta band (average control 0.2 Z-score above baseline, average stress 0.8 Z-score below baseline) (Fig. 1i). Second, we investigated the pairwise temporal correlations among neurons. Exposure to repeated stress prevented the increase in correlations, which naturally occurred in the control group as a function of time (Fig. 1j), for PNs with both lower and higher activity rates (Fig. 1k). Interestingly, the pairwise correlation values per mouse between control and stress groups became significant only after 5 days of repeated stress (Fig. 1l). Third, we looked at measures often used in network theory to describe the structure of networks of interacting elements. To this aim, we generated co-activation networks whose nodes were the PNs and whose edges—or functional connectivity strengths—were the positive values of pairwise temporal correlation between neurons (Supplementary Fig. 4c). With this method we found that repeated stress exposure increased the propensity of higher activity rate neurons to cluster into modules (Modularity, Supplementary Fig. 4d–f) and the propensity of neurons to interact with neurons of similar connectivity degree (Assortativity, Supplementary Fig. 4g–i) mostly due to neurons with higher activity rates (Supplementary Fig. 4f, i).

Altogether, these data show that while repeated stress exposure increases the amount of activity in dCA1, it impairs the temporal coordination of this activity leading to relative decoupling of higher and lower activity neurons.

### Repeated stress exposure increases the number of ensembles but lowers the size and reactivation strength of these ensembles

**Fig. 2:**
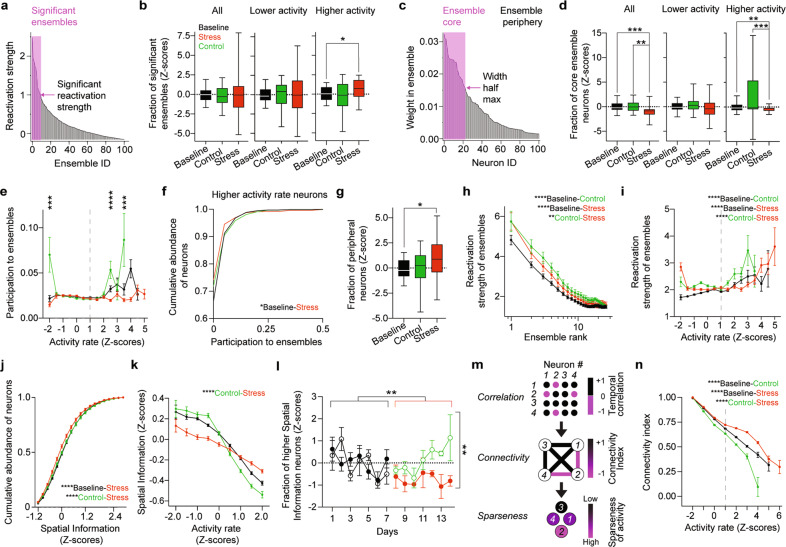
Exposure to repeated stress impairs temporal and spatial codes of PNs in dCA1. **a** Neuronal ensembles (one mouse, one exploration session) sorted according to their normalized reactivation strengths. The arrow denotes the threshold for reactivation strength = 1, the pink rectangle indicates the significant ensembles corresponding to reactivation strength > 1. **b** Stress exposure did not change the fraction of significant ensembles among all (left) and lower activity neurons (middle) but significantly increased the fraction of significant ensembles among higher activity neurons (right). All: *p*_B-C_ > 0.99, *p*_B-S_ > 0.99, *p*_C-S_ > 0.99; *n*_B_ = 84, *n*_C_ = 28, *n*_S_ = 45. Lower activity: *p*_B-C_ > 0.99, *p*_B-S_ > 0.99, *p*_C-S_ > 0.99; *n*_B_ = 83, *n*_C_ = 28, *n*_S_ = 43. Higher activity: *p*_B-C_ > 0.99, *p*_B-S_ = 0.03, *p*_C-S_ = 0.17; *n*_B_ = 50, *n*_C_ = 15, *n*_S_ = 32. **c** Neurons (one mouse, one exploration session) sorted according to their weights within an ensemble. The arrow indicates the threshold that demarcates the ensemble’s core (pink rectangle). **d** Stress exposure decreased the size of ensembles’ cores for all neurons (left: *p*_B-C_ > 0.99, *p*_B-S_ < 0.0001, *p*_C-S_ = 0.0017; *n*_B_ = 87, *n*_C_ = 24, *n*_S_ = 38), mainly due to higher activity neurons. Lower activity: *p*_B-C_ = 0.84, *p*_B-S_ = 0.38 *p*_C-S_ = 0.09; *n*_B_ = 83, *n*_C_ = 28, *n*_S_ = 40. Higher activity: *p*_B-C_ = 0.25, *p*_B-S_ = 0.0002, *p*_C-S_ = 0.09; *n*_B_ = 80, *n*_C_ = 26, *n*_S_ = 39. Box plots: medians and quartiles of mean fraction of significant ensembles **b** or of neurons participating in ensembles’ cores **d** distributions per mouse per session. **e** Participation of high activity rate neurons in ensembles’ cores increased with time, but stress exposure prevented this increase (*p*_-2_ < 0.001, *p*_-1.5, -1, -0.5, 0, 0.5, 1, 1.5, 2_ > 0.13, *p*_2.5_ < 0.0001, *p*_3_ = 0.96, *p*_3.5_ < 0.0001; *n*_-2_ > 68, *n*_-1.5, -1, -0.5_ > 702, *n*_0_ > 2461, *n*_0.5, 1, 1.5, 2_ > 285, *n*_2.5_ > 75, *n*_3_ > 18, *n*_3.5_ > 5); Pairwise, two-tailed *t*-tests between control and stress per activity rate bin. *P*-values corrected for multiple comparisons with Holm-Šidák Method. Circles: mean ensemble participation index binned over activity rates. Error bars: s.e.m. Dashed line indicates the threshold for neurons with higher activity rates. **f** Stress exposure decreased the mean participation of higher activity rate neurons to ensembles’ cores (*p*_B-C_ > 0.99, *p*_B-S_ = 0.031, *p*_C-S_ = 0.32; *n*_B_ = 4445, *n*_C_ = 1201, *n*_S_ = 5863). **g** Stress exposure increased the fraction of neurons in ensembles’ peripheries (*p*_B-C_ > 0.99, *p*_B-S_ = 0.046, *p*_C-S_ = 0.28; *n*_B_ = 40, *n*_C_ = 28, *n*_S_ = 35). Box plots: medians and quartiles of mean fraction of neurons participating in ensemble’s periphery distributions per mouse per session. **h** Reactivation strength of ensembles increased with time, but to a lower extent upon stress exposure (*p*_B-C_ < 0.0001, *p*_B-S_ < 0.0001, *p*_C-S_ = 0.0072; *n*_B_ = 1462, *n*_C_ = 510, *n*_S_ = 749; Two-Way ANOVA). Circles: mean reactivation strengths of ensembles index ranked according to decreasing values. Error bars: s.e.m. **i** Stress exposure increased the activity rates of neurons participating in ensembles with similar reactivation strengths (*p*_B-C S_ < 0.0001, *p*_B-S_ < 0.0001, *p*_C-S_ < 0.0001; *n*_B_ = 25230, *n*_C_ = 9575, *n*_S_ = 11668). Circles: mean reactivation strengths of the ensembles in which each neuron took part binned over activity rates. Error bars: s.e.m. Dashed line indicates the threshold for neurons with higher activity rates. **j** Stress exposure decreased the amount of SI per neuron (*p*_B-C_ = 0.38, *p*_B-S_ < 0.0001, *p*_C-S_ < 0.0001; *n*_B_ = 1152, *n*_C_ = 396, *n*_S_ = 540). **k** Stress exposure decreased the SI in neurons with lower activity rate and increased the SI in neurons with higher activity rate (*p*_B-C_ = 0.21, *p*_B-S_ = 0.074, *p*_C-S_ < 0.0001; *n*_B_ = 9575, *n*_C_ = 25,230, *n*_S_ = 1168). Circles: mean SI binned over activity rates. Error bars: s.e.m. **l** Stress exposure decreased the fraction of neurons with higher spatial information (*p*_Bcontrol-Bstress_ > 0.99, p_Bcontrol-C_ > 0.99, *p*_Bstress-S_ = 0.0011, *p*_C-S_ = 0.0014; *n*_Bcontrol_ = 22, *n*_Bstress_ = 37, *n*_C_ = 26, *n*_S_ = 29). Circles: mean fraction of higher SI neurons per mouse per session. Solid symbols, stress group. Empty symbols, control group. Error bars: s.e.m. **m** Schematic description of the transformation from correlation adjacency matrices to connectivity networks and their meaning in terms of temporal sparseness of activity. **n** Stress exposure increased co-activity and thus prevented the increase in sparseness of activity of dCA1 PNs (*p*_B-C S_ < 0.0001, *p*_B-S_ < 0.0001, *p*_C-S_ < 0.0001; *n*_B_ = 14303, *n*_C_ = 6054, *n*_S_ = 9026). Circles: mean connectivity index binned over activity rates. Error bars: s.e.m. Dashed line indicates the threshold for neurons with higher activity rates. **b**, **d**, **f**, **g**, **l** Kruskal–Wallis test, *p*-values adjusted after Dunn’s corrections for multiple comparisons. **i**, **j**, **k**, **n** Two-way ANOVA, *p*-values adjusted after Tukey’s corrections for multiple comparisons.

Ensemble activity is a form of temporal organization thought to underpin information representation [43, 44] and memory formation [45–48]. We used principal component analysis to extract activity patterns corresponding to neural ensembles and defined significant ensembles as ensembles whose encoding strength during a session was ≥1 [37] (Fig. 2a). Repeated stress exposure increased the fraction of significant ensembles comprising only higher activity rate neurons (Fig. 2b and Supplementary Fig. 5a). We demarcated the ensembles’ cores from their peripheries by sorting neurons according to their weights and used the half maximum width of the weight’s distribution as a threshold (Fig. 2c). Repeated stress exposure decreased the size of ensembles’ cores, mainly due to the contribution of higher activity rate neurons (Fig. 2d and Supplementary Fig. 5b). While participation of neurons to ensembles’ cores increased in control groups, stress exposure prevented this increase mostly by decreasing the mean participation of higher activity rate neurons to ensembles’ cores (Fig. 2e, f and Supplementary Fig. 5c) and increasing the fraction of neurons in ensembles’ peripheries (Fig. 2g). The ensemble’s encoding strength increased as a function of time (or familiarity with the arena), but the magnitude of this increase was lower during repeated stress exposure (Fig. 2h). Moreover, neurons participating in ensembles with encoding strengths similar to control showed activity rates higher than control upon stress exposure (Fig. 2i).

In summary, these results reveal that repeated stress exposure impairs the temporal organization of dCA1 activity patterns by leading to smaller and less prominent neuronal ensembles.

### Repeated stress exposure impairs spatial coding in dCA1

Overall, exposure to repeated stress impact temporal coding of CA1 PNs, we thus wondered whether it also affects spatial coding. To this aim we calculated the amount of Spatial Information (SI) [38] per neuron in a total of 25230, 9575 and 11668 neurons during baseline, control and stress periods respectively. Repeated stress exposure significantly decreased the amount of SI (Fig. 2j). During baseline lower activity rates were associated with higher SI and higher activity rates were associated with lower SI, with time (control group) this sigmoidal relationship sharpened trending towards increase of SI in lower activity neurons and decrease of SI in higher activity neurons. Repeated stress exposure, however, significantly decreased SI in lower activity neurons and increased SI in higher activity neurons (Fig. 2k). Stress exposure also affected the relationship between SI and the participation to population bursts in an analogous fashion: by decreasing the participation of neurons with lower SI and increasing the participation of neurons with higher SI (Supplementary Fig. 5d). Thus, repeated stress exposure impaired the relationship between activity rates, SI and participation in population bursts (Supplementary Fig. 5e).We then investigated the effect of repeated stress exposure on place cell-like activity by focusing on neurons with the highest SI (SI ≥ 2.5 Z-score, Supplementary Fig. 5f). Stress exposure decreased the fraction of high-SI neurons (Fig. 2l) with the difference between control and stress groups becoming highly significant only after 5 days of repeated stress (Supplementary Fig. 5g).

Overall, repeated stress exposure impairs spatial coding in dCA1 by disrupting the relationship between activity rates, SI and participation in population bursts and preventing the increase in place cell-like activity.

### Repeated stress exposure decreases the sparseness of activity in dCA1

Neurons with higher SI show a temporally sparse activity patterns, thus we wondered whether repeated exposure to stress would decrease sparseness of activity alongside to SI in dCA1 PNs. To this aim we calculated a connectivity index for each PN (see Methods) representing the temporal sparseness of the activity of a given PN. Indices closer to 1 indicate neurons being more co-active with other neurons (less sparse activity) and indices closer to -1 indicate neurons being less co-active with other neurons (more sparse activity) (Fig. 2m). Repeated exposure to stress significantly increased co-activity and thus prevented the increase in sparseness of activity of dCA1 PNs, which naturally occurred between the baseline and control epochs (Fig. 2n).

### Long-term tracking of dendritic spines in basal dendrites of dCA1 PNs

**Fig. 3:**
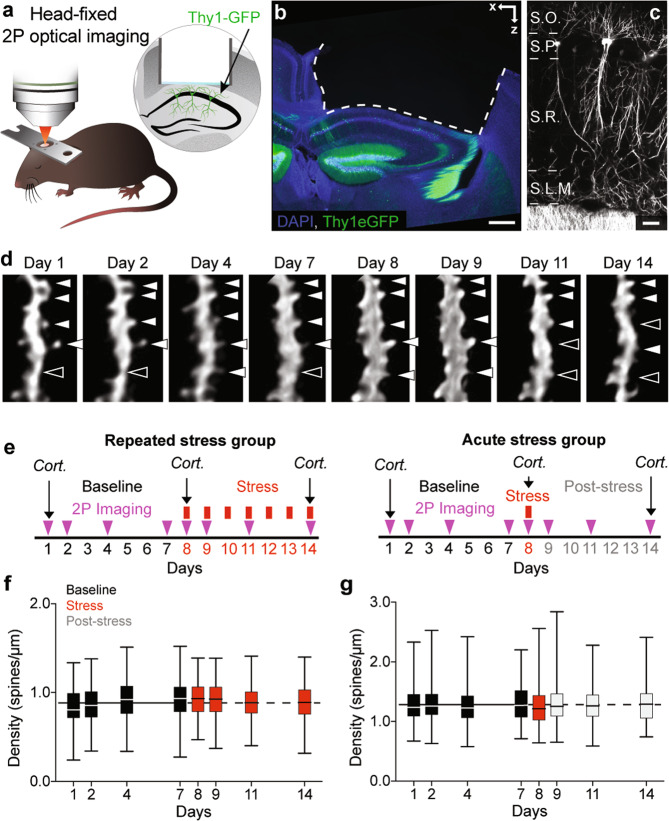
Longitudinal tracking of dendritic spines in dCA1 PNs during stress exposure. **a** Schematic description of 2 P optical imaging of PNs in the dCA1 in a head-fixed anesthetized mouse. **b**, **c** Confocal images of the dorsal hippocampus **b** and dCA1 **c** of an experimental Thy1-GFP mouse. Blue, DAPI and green, GFP **b**. White GFP **c**. S.O. Stratum Oriens, S.P. Stratum Pyramidale, S.R. Stratum Radiatum, S.L.M. Stratum Lacunosum-Molecolare. Scale bars, 200 µm **b** and 20 µm **c**. **d** 2 P time-lapse image sequence showing a basal dendritic segment of a dCA1 PN of a live experimental Thy1-GFP mouse (maximum intensity projection of 3 to 5 Z focal planes each) and dendritic spines (white full triangles, spines present; empty white tringles, spines absent) showing dynamic behavior. Scale bar, 1 µm. **e** Experimental timelines for mice stressed repeatedly (left) or acutely (right). Cort. indicates measurements of plasma Corticosterone levels. **f**, **g** Repeated **f** and acute **g** stress exposure did not affect spine densities. **e** *p*_8, 9,11, 14_ > 0.22, *n*_B_ = 248, *n*_8, 9, 11, 14_ = 114; **f** *p*_8,9,11,14._ > 0.82; *n*_B_ = 352, *n*_8, 9, 11, 14_ = 88. Kruskal–Wallis tests against pooled baseline distribution, *p*-values adjusted after Dunn’s multiple comparisons. Box plots: medians and quartiles of spine densities distributions per dendrite. Black solid and dashed horizontal lines: mean density of spines during baseline.

While activity rate increased immediately after the onset of repeated stress (Fig. 1e), correlation of activity (Fig. 1j, k) and spatial coding (Fig. 2l) became significantly different from control animals only after 5 days of repeated stress exposure. This suggests that sustained hyperactivity leads to higher order effects on temporal and spatial coding. As hyperactivity can lead to homeostatic scaling of synapses [49], we pondered whether decrease in synaptic connectivity could mediate the effects of prolonged hyperactivity on temporal and spatial coding in the dCA1 during repeated stress exposure. We thus investigated synaptic connectivity in mice undergoing the same paradigm of repeated multimodal stress exposure, by using deep-brain 2 P optical imaging [50] (Fig. 3a). As a proxy for excitatory synaptic connectivity, we imaged dendritic spines in mice expressing cytoplasmic GFP in a random subset of dCA1 PNs under the control of the Thy1 promoter [51] (Fig. 3b, c). We tracked dynamics of dendritic spines in the basal dendrites of dCA1 PNs longitudinally for 2 weeks (Fig. 3d). After 1 week of baseline imaging, mice were exposed to multimodal stress for 2 h, every day, for 7 days. Stress exposure ended at least 2 h prior to imaging (Repeated stress group, Fig. 3e). In addition, to control for repeated imaging and to compare the effects of repeated and acute stress, we imaged a second group of Thy1-GFP mice undergoing a single exposure to 2 h multimodal stress followed by 6 days of recovery (Acute stress group, Fig. 3e). We tracked 114 (Repeated stress group) and 88 (Acute stress group) basal dendritic segments throughout the experiment and imaged an average of 3857 (±67 s.e.m.), 3927 (±41 s.e.m.) (Repeated stress group) and 3581 (±10 s.e.m.), 3540 (±27 s.e.m.) (Acute stress group) spines per session during baseline and stress respectively. The density of spines was stable during the baseline period and we found no significant changes in spine density in either the Repeated and Acute stress groups (Fig. 3f, g).

We detected a sustained increase in the levels of plasma corticosterone in the Repeated stress group and only a transient increase in the Acute stress group (Supplementary Fig. 6a, b).

### Repeated stress exposure leads to immediate decrease in spinogenesis followed by spine loss in dCA1 PNs

**Fig. 4:**
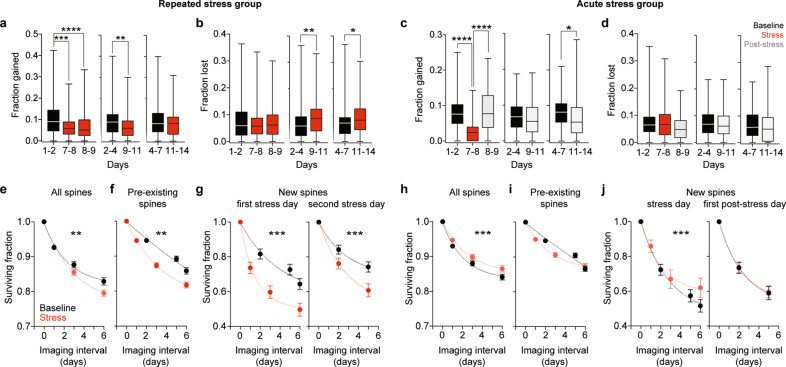
Repeated and acute stress exposures have different effects on gain, loss and survival of dendritic spines in dCA1 PNs. **a** Repeated stress exposure decreased the fraction of spines gained in the 1–4-day interval after stress onset (*p*_7–8_ = 0.0001, *p*_8–9_ < 0.0001, *p*_9–11_ = 0.0022, *p*_11–14_ = 0.7466, *n* = 124). **b** Repeated stress exposure increased the fraction of spines lost in the 4-to-7-day interval after stress onset (*p*_7–8_ = 0.7144, *p*_8–9_ > 0.9999, *p*_9–11_ = 0.0024, *p*_11–14_ = 0.0166, *n* = 124). **c** Acute stress decreased the fraction of spines gained immediately after stress exposure (*p*_7–8_ < 0.0001, *p*_8–9_ > 0.9999, *p*_9–11_ = 0.3483, *p*_11–14_ = 0.0182, *n* = 88). **d** Acute stress had no effect on spine loss (**f**, *p*_7–8_ > 0.9999, *p*_8–9_ = 0.3263, *p*_9–11_ = 0.8860, *p*_11–14_ = 0.2665, *n* = 88). Wilcoxon matched pairs signed ranks tests to 1–2, 2–4 and 4–7 respectively, *p*-values adjusted after Dunn’s correction for multiple comparisons Box plots: medians and quartiles of the distributions of fractional spine gain **a**, **c** or loss **b**, **d** per dendrite. **e**–**g** Repeated stress exposure decreased the survival of all **e**, pre-existing **f** and new **g** spines detected on the first (left) and second (right) days of stress. *p*_All_ = 0.003, *p*_Pre-existing_ = 0.001, *p*_NewFirstday_ < 0.001, *p*_NewSecondday_ < 0.001, *n*_All_ = 372, *n*_Pre-existing_ = 372, *n*_NewFirstday_ = 312, *n*_NewSecondday_ = 130. **h**–**j** A single exposure to stress increased the survival of all spines (**h**), but it did not affect pre-existing spines (**i**). Increase in spine survival was due to increased survival rate of new spines detected on the day of stress (**j**, left) but not on the following day (**j**, right). *p*_All_ < 0.001, *p*_Pre-existing_ = 0.251, *p*_NewFirstday_ < 0.001, *p*_NewSecondday_ = 0.861, *n*_All_ = 264, *n*_Pre-existing_ = 264, *n*_NewFirstday_ = 177, *n*_NewSecondday_ = 154. Mann–Whitney *U*-test between all non-1 Baseline versus Stress points. Circles: mean surviving fractions per dendrite. Error bars: s.e.m. Curves: single exponential decays fit to the data.

Dendritic spines of dCA1 are dynamic [29, 52] and their persistence correlates to the probability of neurons to become active and to the ability of mice to recall a hippocampal memory task [31]. We thus investigated addition and persistence of spines upon stress exposure. Repeated stress led to immediate decrease in spinogenesis that continued for 4 days during stress exposure (Fig. 4a), and—as a consequence—to protracted reduction in the density of new spines (Supplementary Fig. 7a). Repeated stress also increased the fraction of spines lost but only starting from day 4 of stress exposure (Fig. 4b). By comparison, acute stress led to an immediate decrease in spinogenesis (Fig. 4c) and new spine density (Supplementary Fig. 7b), but this decrease was brief and spinogenesis and new spine density quickly recovered after stress exposure ended. In contrast to repeated stress exposure, acute stress had no noteworthy effect on spine loss (Fig. 4d).

In summary, stress affected spine dynamics proportionally to its duration with repeated stress leading to decrease in spinogenesis followed by sustained increase in spine loss (Supplementary Fig. 7c) and acute stress decreasing spinogenesis only acutely (Supplementary Fig. 7d).

### Repeated and acute stress have opposite effects on stability of spines

Consistent with increased loss of dendritic spines, we found a decrease in spine survival (Fig. 4e) upon repeated stress exposure. Spine survival decreased not only in spines that already existed before the stress event (Fig. 4f) but also in new spines detected on the first (Fig. 4g, left) and second (Fig. 4g, right) days of stress exposure. In stark contrast, a single exposure to stress increased spine survival (Fig. 4h). Interestingly, increased synaptic stability, was not due to increased survival of spines that already existed before the stressful experience (Fig. 4i) but rather to increased survival of new spines detected on the day of stress exposure (Fig. 4j, left). The survival of spines that appeared one day after the stressful experience was not affected (Fig. 4j, right).

Thus, while repeated stress exposure destabilizes spines born both before and after stress onset, acute stress stabilizes specifically only spines born in temporal proximity to the single stress experience.

### Stress impairs learning and recall in Morris water maze spatial memory task

**Fig. 5:**
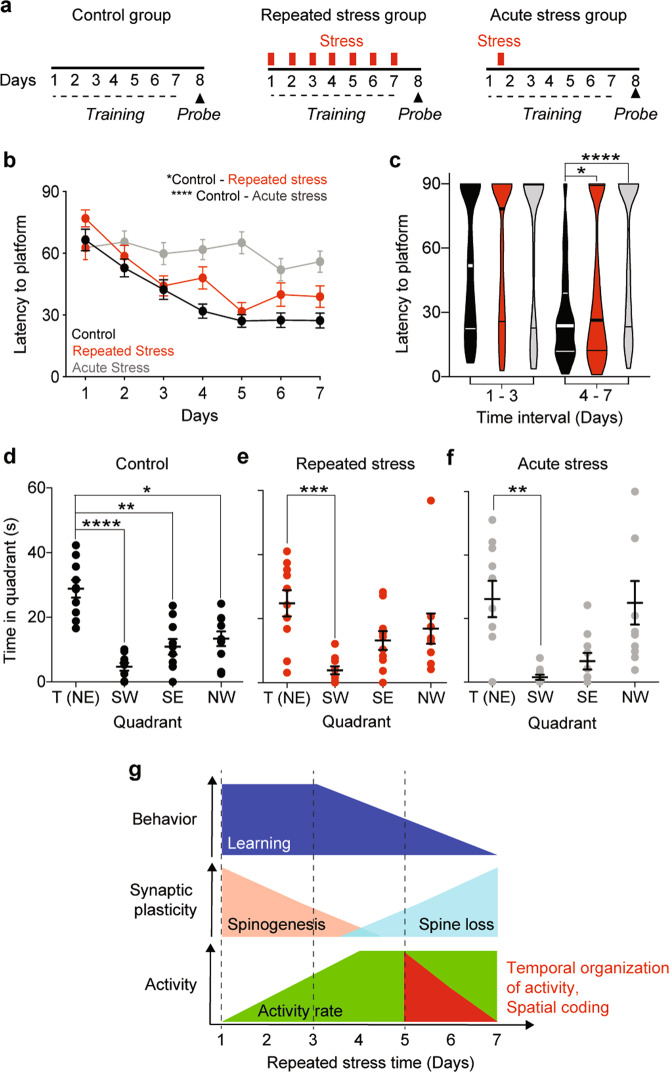
Repeated and acute stress exposure impair learning and recall of a spatial memory task. **a** Experimental timeline for Morris water maze training (dashed lines) and testing (triangles, probe) of Control, Repeated or Acute stress mice groups. **b** Repeated stress during training and acute stress on the first training day, increased latency to reach the hidden platform (*p*_CA_ < 0.0001 and *p*_CR_ = 0.045 l; *n* = 40 per time point; Repeated Measurements ANOVA). Circles: average latency of 10 (4 trials each) mice. Black, Control; red, Repeated; gray, Acute stress groups. **c** Latencies of Repeated (red) and Acute (gray) stress groups differed significantly from the ones of the Control (black) group on days 4–7 (*p*_CA_1–3_ = 0.56 and *p*_CR_1–3_ = 0.91; *p*_CA_4–7_ < 0.0001 and *p*_CR_4–7_ = 0.034; *n*_1–3_ = 120 per group, *n*_4–7_ = 160 per group). Violin plots: median and quartiles of the distributions of latencies pooled over days 1–3 or 4–7 for Control (black), Repeated (red) or Acute (gray) stress groups. **d** Control mice spent significantly more time in the target quadrant (NE), during the probe trial (*p*_SW_ < 0.0001, *p*_SE_ = 0.004, *p*_NW_ = 0.036; *n* = 10 animals per quadrant). **e** Mice stressed repeatedly during training spent significantly less time only in the quadrant opposite to the target quadrant during probe trial (*p*_SW_ =0.002, *p*_SE_ = 0.29, *p*_NW_ = 0.68; *n* = 10 animals per quadrant). **f** Mice stressed acutely on the first training day spent significantly less time only in the quadrant opposite to the target quadrant during probe trial (*p*_SW_ = 0.003, *p*_SE_ = 0.87, *p*_NW_ > 0.999_;_ *n* = 10 animals per quadrant). Circles: time per quadrant per mouse. Black, Control; red, Repeated and gray, Acute stress groups. Horizontal lines: means ± s.e.m. **c**–**f** Kruskal–Wallis test, *p*-values adjusted after Dunn’s corrections for multiple comparisons. **g** Summary and temporal sequence of stress-induced impairments in dCA1 PNs connectivity and activity patterns as well as behavior.

Dynamics of dendritic spines have been associated with the ability to learn [21–24, 31, 53]. We thus tested the ability of mice undergoing repeated or acute multimodal stress to learn and recall a hippocampal dependent spatial task. To this aim, we trained three groups of mice to learn the position of an escape platform in the classic hippocampal-dependent Morris water maze learning task under control, repeated, or acute stress conditions (Fig. 5a). Stressed animals showed impaired learning, as measured by the decline in escape latency to the hidden platform. Surprisingly, the Acute stress group showed stronger impairment in learning with almost no decrease in latency over the training period (Fig. 5b gray curve). Interestingly, the learning curves of repeatedly stressed and control animals overlapped during the first 3 days of training and became significantly different only starting from day 4 of stress (Fig. 5c). While control animals spent significantly more time in the target quadrant than all other quadrants (Fig. 5d), stressed animals significantly avoided only the quadrant opposite to the target quadrant but not the neighboring quadrants (Fig. 5e, f).

These results show differential effect of acute and repeated stress in spatial learning and confirm that stress has lasting effects on the ability of animals to learn and recall spatial memories.

## Discussion

By combining WFHM and 2 P deep-brain optical imaging we investigated the link between hyperactivity, synaptic connectivity, temporal structure of activity and spatial coding of dCA1 PN’s in mice undergoing stress exposure. We characterized network- and synaptic-level endophenotypes associated with stress and described a temporal sequence of events suggesting a role for decreased synaptic connectivity as a mediator between hyperactivity and impaired activity patterns and spatial coding in dCA1.

### Neuronal activity increases with stress exposure but it is disorganized

Repeated stress exposure led to increased activity in dCA1 PNs, in line with previous reports [26, 39, 54], but the temporal organization of this activity significantly differed from control. First, while repeated exploration of the same environment led to increased theta power and pairwise correlation of neuronal activity (consistent with previous work [55, 56]), four consecutive exposures to stress blunted this increase. Second, repeated stress exposure decreased the power of underlying oscillations in the Theta range. This is of special importance in the hippocampus, as oscillations in the Theta range have been implicated in local information processing [38, 57–59], long-range communication to neocortical areas [55, 60] and synaptic plasticity [61]. Third, neurons with higher activity rates participated less in bursts and segregated into modules but were excluded from the cores of ensembles. This has import because, while highly active neurons are a smaller fraction of all active neurons [62], they tend to be stably active through time [63], can be preferentially recruited into engrams [64, 65] and possibly dominate information transfer in the hippocampus and other cortical regions [62, 66]. Fourth, repeated stress exposure decreased the size of ensembles and prevented increase in the ensemble’s reactivation strength. Pyramidal neurons in CA1 can show sequential activity within a few hundred milliseconds giving rise to neuronal ensembles [38, 67, 68]. Ensemble activity is a form of temporal organization thought to underpin information representation [43, 44] and memory formation [45–48]. In addition, the strength of reactivation of these neuronal ensembles is important for hippocampal learning and recall [48, 69]. Altogether, stress-induced weakening of neuronal activity synchronization, changes in activity patterns of neurons with higher activity rates and disruption of the temporal structure of neuronal activity might prejudice information flow and contribute to learning impairments.

### Repeated stress exposure impairs spatial coding of dCA1 PNs

We also found not only a reduction of the average amount of SI in dCA1 PNs but a specific decrease in the abundance of neurons with higher SI—which can be interpreted as place cells—over control from 5 days of repeated stress onwards, mirroring the decrease in correlation of activity. Interestingly, we uncovered a noteworthy impairment of spatial coding efficiency. In fact, while repeated exploration of the arena led to more SI being encoded with lower activity rates which is a more energy-efficient way to encode space, repeated stress led to less spatial information being coded with higher activity rates which is comparatively a less energy-efficient spatial coding.

### Decreased spinogenesis precedes increased spine loss upon exposure to repeated stress

Longitudinal imaging enabled us to establish two different effects on synaptic dynamics with different onsets. First, a decrease in spinogenesis which appeared immediately after a single exposure to stress and lasted for up to 4 days of repeated stress exposure. This might depend on non-genomic effects of glucocorticoids and corticotropin releasing factor [4] and is consistent with the fact that stress decreases LTP [12, 14, 70] and LTP induction promotes spinogenesis [71, 72]. Decreased spinogenesis, however, was transient and recovered to baseline levels the day after acute stress or after 4 days of repeated stress exposures, suggesting the presence of compensatory mechanisms working even under sustained stress conditions. Second, a loss in spines which became apparent after 4 days of repeated stress exposure but not after a single stress exposure. Thus, while decrease in spinogenesis might represent the first response to counteract the surge in activity linked to stress, only sustained exposure to stress causes actual synaptic loss—possibly due to toxic or homeostatic effects of the sustained hyperactivity -. These results are in line with recent work reporting loss of inputs in the posterior parietal cortex upon a similar repeated stress paradigm [40].

Previous work has shown a small but significant decrease in dCA1 spine density upon stress exposure [9, 73], thus it might seem surprising that the changes in dynamics we report here do not lead to apparent changes in spine density. This discrepancy might be due to many factors: (i) intrinsic differences between the different animal models used—mice *vs*. rats [73]—, (ii) the dCA1 layer analyzed—basal *vs*. apical [9]—, (iii) the different methods employed to detect spines—2 P *vs*. confocal and wide field optical imaging—or (iv) the total duration of stress exposure. In fact, decreased spine density in the apical dendrites of dorsal CA1 was evident after 3 weeks of daily restraint stress [9], a stress exposure duration three times longer than ours.

### Impairment in synaptic connectivity follows hyperactivity but precedes weakening of temporal organization of activity and impairment in spatial coding

A comparison of the onset of activity and structural synaptic impairments after beginning of repeated stress exposure reveals a precise temporal sequence of events. Increase in activity and decrease in spinogenesis occurred immediately, increase in spine loss and impairment in learning occurred at three to 4 days, and weakening in the temporal structure of activity and spatial coding happened at 5 days after repeated stress onset (Fig. 5G). This temporal sequence suggests that stress-induced sustained increase in activity leads to loss of structural connectivity which in turn leads to weakening of temporal structure of activity and spatial coding. However, we cannot exclude that all these changes occur independently and further work is needed to demonstrate causality. Specifically, it will be important to manipulate synaptic stability to either rescue changes in temporal or spatial coding and behavior during repeated stress exposure of to mimic them in the absence of stress.

### Stress-induced stabilization of new spines in dCA1 might support learning of stress-related events

Acute and repeated stress exposure showed opposite effects on the stability of excitatory synaptic connectivity. While repeated stress exposure decreased overall spine survival, acute stress increased the survival of the spines born in temporal vicinity to stress exposure. Such stabilization could depend on stress-specific regulation of adhesion molecules such as NCAM or L1 [74], on increased trafficking of AMPA receptors due to non-genomic action of glucocorticoids [75, 76]. Notably, increase in stability was more prominent in spines born in temporal vicinity to the exposure to the stressful event. By analogy to the neocortex—where stabilization of new dendritic spines supports acquisition of new memories [21–24]—increase in stability of the spines born in temporal proximity to stress exposure might be a cellular mechanism supporting learning of information related to the stressful event [77]. Our findings that acute stress leads to a more pronounced impairment in learning than repeated stress support this hypothesis. If an acute stressful experience stabilizes specifically the dCA1 spines born in temporal proximity to this experience, these synapses could mediate the association between the acute stress negative experience and another experience occurring close in time, such as navigation in the water maze. This negative association could interfere with future similar experiences, thus leading to a lasting and potent inhibition of learning, consistent with what we find. Further experiments are necessary to confirm the causal role of dCA1 spines in the formation of this association and to test whether acute stress exposure would affect specifically a task learned in temporal association with the stressful event but not translate to a different task learned before or after it [77].

## Supplementary information

Supplementary Material

## Data Availability

The analysis code can be found on Github. All original raw data will be made available by the corresponding author upon request.
