# Supplementary material for: Repeated stress exposure leads to structural synaptic instability prior to disorganization of hippocampal coding and impairments in learning
Source: Transl Psychiatry. 2022 Sep 12;12:381. doi: 10.1038/s41398-022-02107-5 (PMC9468341; doi:10.1038/s41398-022-02107-5)
Supplement: Supplementary file 1 — Supplementary Material [file 41398_2022_2107_MOESM1_ESM.pdf]

Repeated stress exposure leads to structural synaptic instability prior to disorganization of hippocampal coding and impairments in learning

Alireza Chenani[1*], Ghabiba Weston[1,2*], Alessandro F. Ulivi[1,3], Tim P. Castello-Waldow[1], Rosa-Eva Huettl[1], Alon Chen[1,2,4] and Alessio Attardo[1,2,3]

[1] Max Planck Institute of Psychiatry, 80804, Munich, Germany.

[2] Graduate School of Systemic Neurosciences GSN-LMU, 82152, Munich, Germany.

[3] Leibniz Institute of Neurobiology, 39118 Magdeburg, Germany

[4] Weizmann Institute of Science, 76100, Rehovot, Israel.

[*] Equal contribution

Corresponding author:

Alessio Attardo

Leibniz Institute of Neurobiology, 39118 Magdeburg, Germany

Telephone: 0049 391 626393371

E-Mail: alessio.attardo@lin-magdeburg.de

SUPPLEMENTARY FIGURES AND CAPTIONS

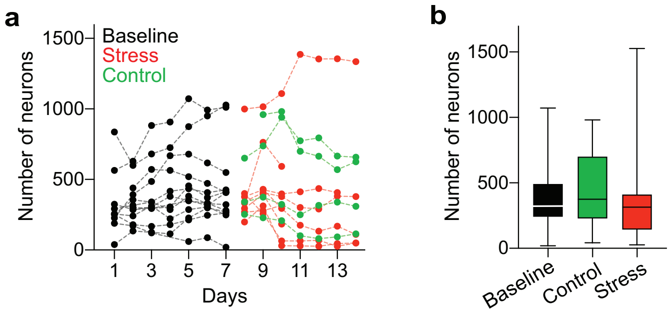

Supplementary Figure 1 | The number of neurons recorded by WFHM optical imaging is stable.

(**a, b)** The number of neurons imaged by WFHM microscopy did not change through baseline, control and stress periods ($p_{B\text{-}C} = 0.83$, $p_{B\text{-}S} > 0.9999$, $p_{C\text{-}S} = 0.27$; $n_B = 106$, $n_C = 35$, $n_S = 60$; Kruskal-Wallis test, p values adjusted after Dunn's corrections for multiple comparisons).

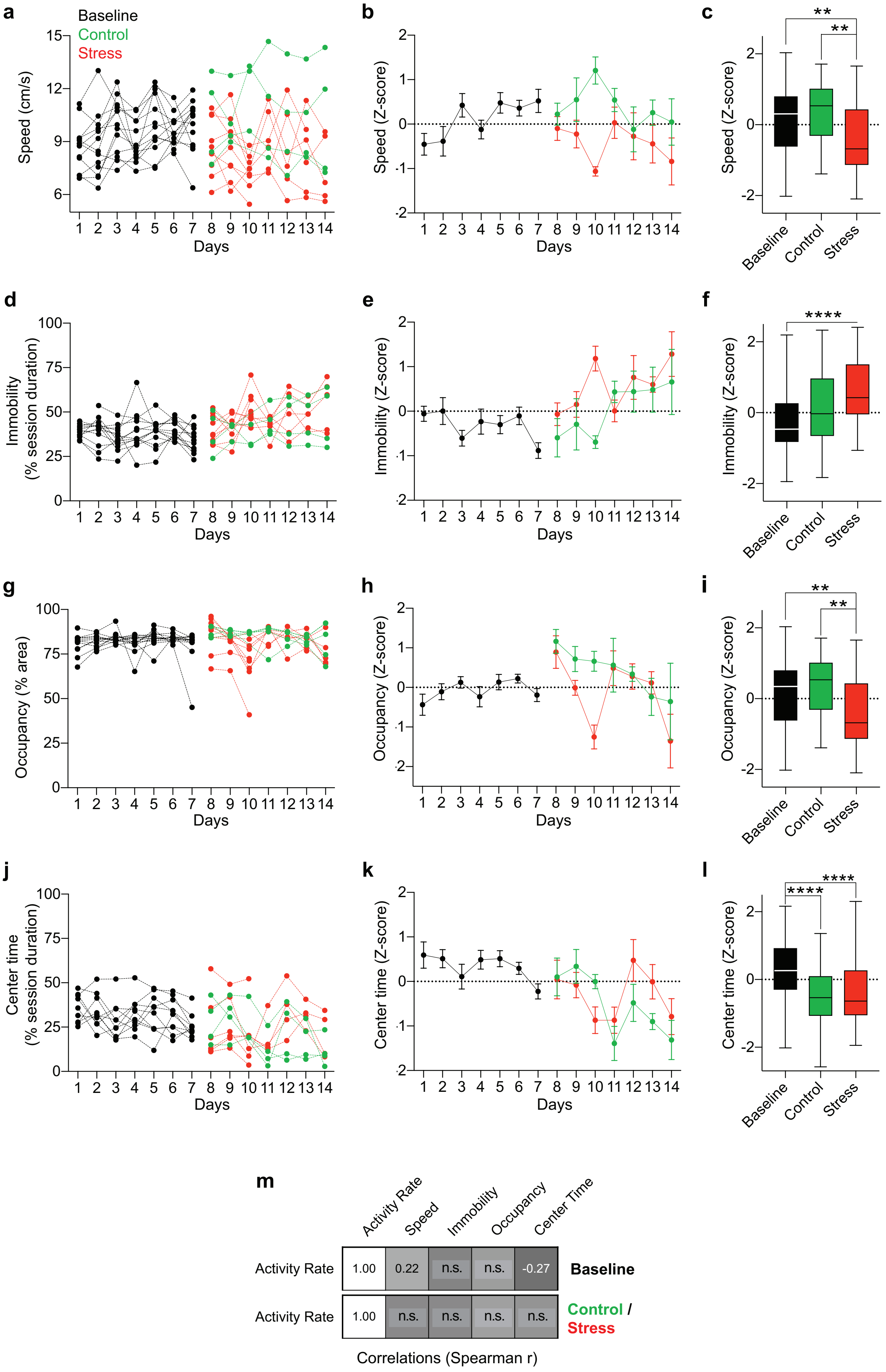
a
Baseline
Control
Stress
Speed (cm/s)
Days
b
Speed (Z-score)
Days
c
Speed (Z-score)
Baseline
Control
Stress
**
**
d
Immobility
(% session duration)
Days
e
Immobility (Z-score)
Days
f
Immobility (Z-score)
****
Baseline
Control
Stress
g
Occupancy (% area)
Days
h
Occupancy (Z-score)
Days
i
Occupancy (Z-score)
**
**
Baseline
Control
Stress
j
Center time
(% session duration)
Days
k
Center time (Z-score)
Days
l
Center time (Z-score)
****
****
Baseline
Control
Stress
m
Activity Rate
Speed
Immobility
Occupancy
Center Time
Activity Rate 1.00 0.22 n.s. n.s. -0.27 Baseline
Activity Rate 1.00 n.s. n.s. n.s. n.s. Control / Stress
Correlations (Spearman r)

Supplementary Figure 2 | Repeated stress impairs several aspects of free navigation in a circular arena.

(**a - c**) Repeated stress decreased average speed during free exploration of the arena ($p_{B-C} = 0.77$, $p_{B-S} = 0.0048$; $p_{C-S} = 0.0023$; $n_B = 90$, $n_C = 27$, $n_B = 90$; $n_S = 50$).

(**d - f**) Repeated stress increased immobility during free exploration of the arena ($p_{B-C} = 0.20$, $p_{B-S} < 0.0001$; $p_{C-S} = 0.22$; $n_B = 90$, $n_C = 27$, $n_B = 90$; $n_S = 50$).

(**g - i**) Repeated stress decreased average occupancy of the arena ($p_{B-C} = 0.77$, $p_{B-S} = 0.0048$; $p_{C-S} = 0.0023$; $n_B = 90$, $n_C = 27$, $n_B = 90$; $n_S = 50$; Kruskal-Wallis test, p values adjusted after Dunn's corrections for multiple comparisons).

(**j - l**) Repeated stress did not affect permanence in the center of the arena ($p_{B-C} = 0.001$, $p_{B-S} < 0.0001$; $p_{C-S} > 0.999$; $n_B = 90$, $n_C = 27$, $n_B = 90$; $n_S = 43$)

Kruskal-Wallis test, p values adjusted after Dunn's corrections for multiple comparisons.

(**m**) Spearman correlations between average Activity rates and open field navigation parameters of all groups during the Baseline (top) or Control / Stress (bottom) epochs. n.s.= not significant.

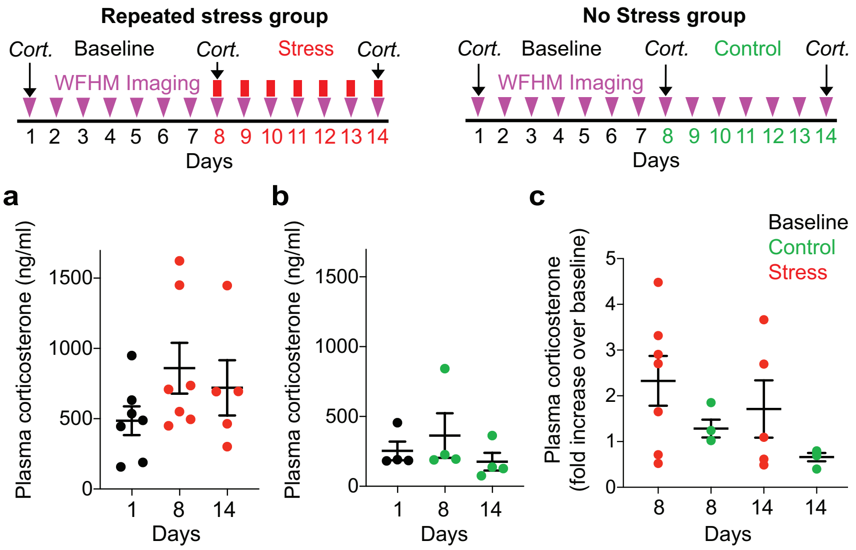

Supplementary Figure 3 | Blood corticosterone levels in mice imaged with WFHM microscopes upon repeated stress exposures

(**a - c**) The blood levels of corticosterone did not significantly increase upon repeated stress exposures (a: $p_{1\text{-}8}$ = 0.12, $p_{1\text{-}14}$ = 0.70, n_1 = 7, n_8 = 7, n_{14} = 5; b: $p_{1\text{-}8}$ = 0.31, $p_{1\text{-}14}$ = 0.31, n_1 = 4, n_8 = 4, n_{14} = 4), although several stressed animals showed a bigger fold increase in the levels of corticosterone in their blood compared to control group (c: $p_{S\text{-}C,\,8}$ > 0.9999, $p_{S\text{-}C,\,14}$ = 0.43; n_8 = 11, n_{14} = 9; Kruskal-Wallis test, p values adjusted after Dunn's corrections for multiple comparisons).

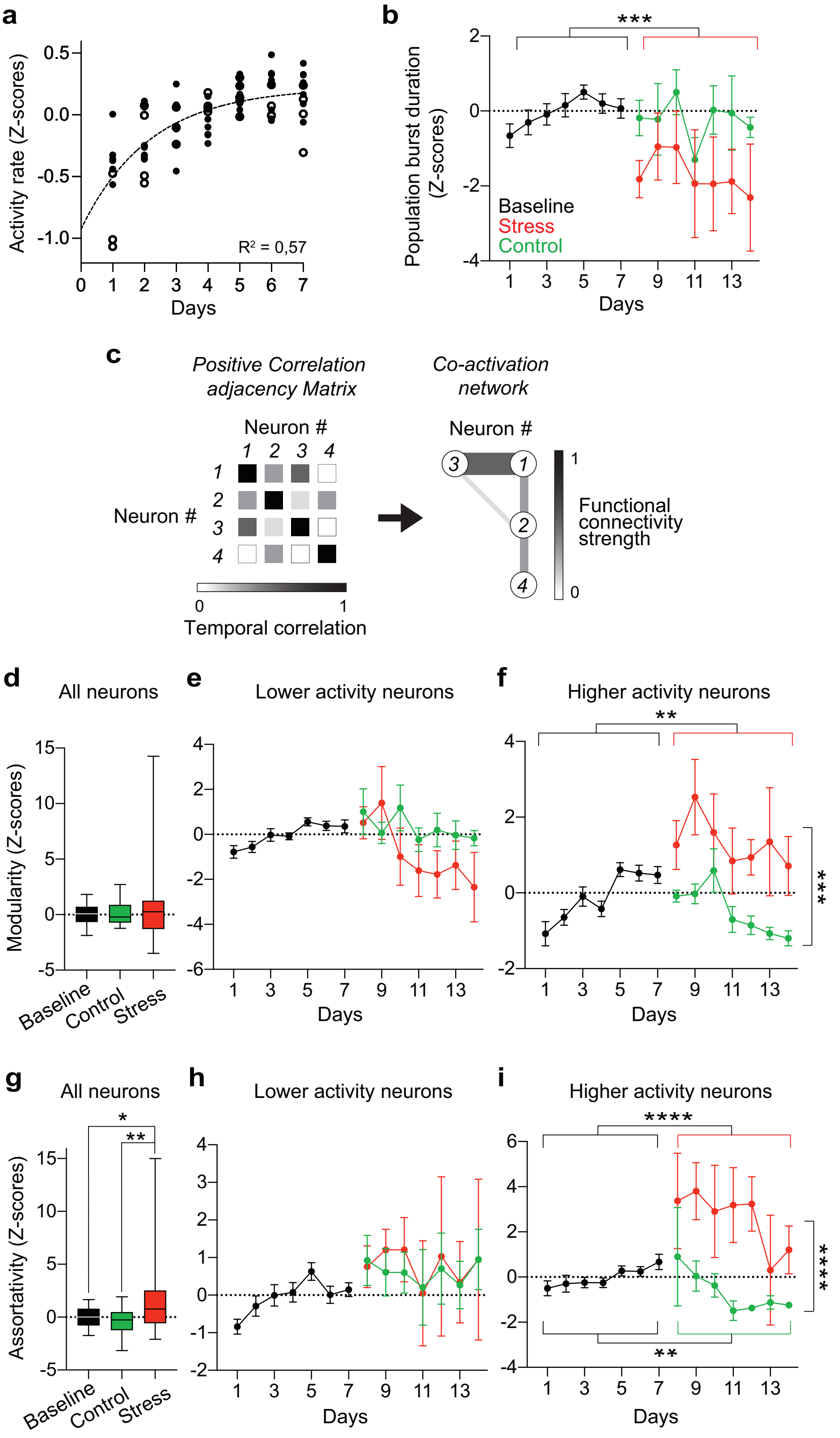

Supplementary Figure 4 | Exposure to repeated stress affects population burst rate and duration and leads to co-activation networks with more modular and assortative topology

(**a**) During baseline activity rates increased in both prospective Control and Stress groups (p_{time} <0.0001, p_{Group} = 0.067; n = 86; Two-way ANOVA) and reached plateau after day 5 (Plateau at Y = 0.224; Single exponential fit to all baseline data; R^2 = 0.567).

(**b**) Stress exposure decreased the duration of the population bursts (p_{B-C} > 0.99, p_{B-S}= 0.0012, p_{C-S}= 0.19; n_B = 86, n_C = 27, n_S = 50)

(**d**) Schematic description of the transformation from positive correlation adjacency matrices to co-activity networks.

(**e - g**) Stress exposure did not affect modularity of networks comprising all neurons (e) and lower activity rate neurons (f) but significantly increased modularity of the network comprising only higher activity neurons (g). e: p_{B-C} > 0.99, p_{B-S} > 0.99, p_{C-S} > 0.99; n_B = 86, n_C = 28, n_S = 49. f: p_{B-C}= 0.99, p_{B-S} = 0.12, pC-S= 0.20; n_B = 86, n_C= 28, n_S= 49. g: p_{B-C}= 0.14, p_{B-S} = 0.009, p_{C-S} = 0.0001; n_B = 79, n_C = 25, n_S = 47.

(**i - j**) Stress exposure increased assortativity of the network comprising all neurons (h) mostly due to increased assortativity of the network comprising only higher activity neurons (i, j). h: p_{B-C} = 0.65, p_{B-S} = 0.022, p_{C-S} = 0.005; n_B = 86, n_C= 85, n_S = 49. i: p_{B-C}= 0.18, p_{B-S} = 0.62, p_{C-S}> 0.99; n_B= 86, n_C = 28, n_S = 49. j: p_{B-C}= 0.006, p_{B-S} < 0.0001, p_{C-S} < 0.0001; n_B = 79, n_C= 25, n_S = 47.

(**b, d – i**), Kruskal-Wallis test, p values adjusted after Dunn's corrections for multiple comparisons.

(**b, e, f, h, i**), Circles: mean population bursts duration (b) modularity (e, f) or assortativity (h, i) per mouse. Error bars: s.e.m.

(**d, g**) Box plots: medians and quartiles of the distributions of mean modularity (d) or assortativity (g) per mouse per session.

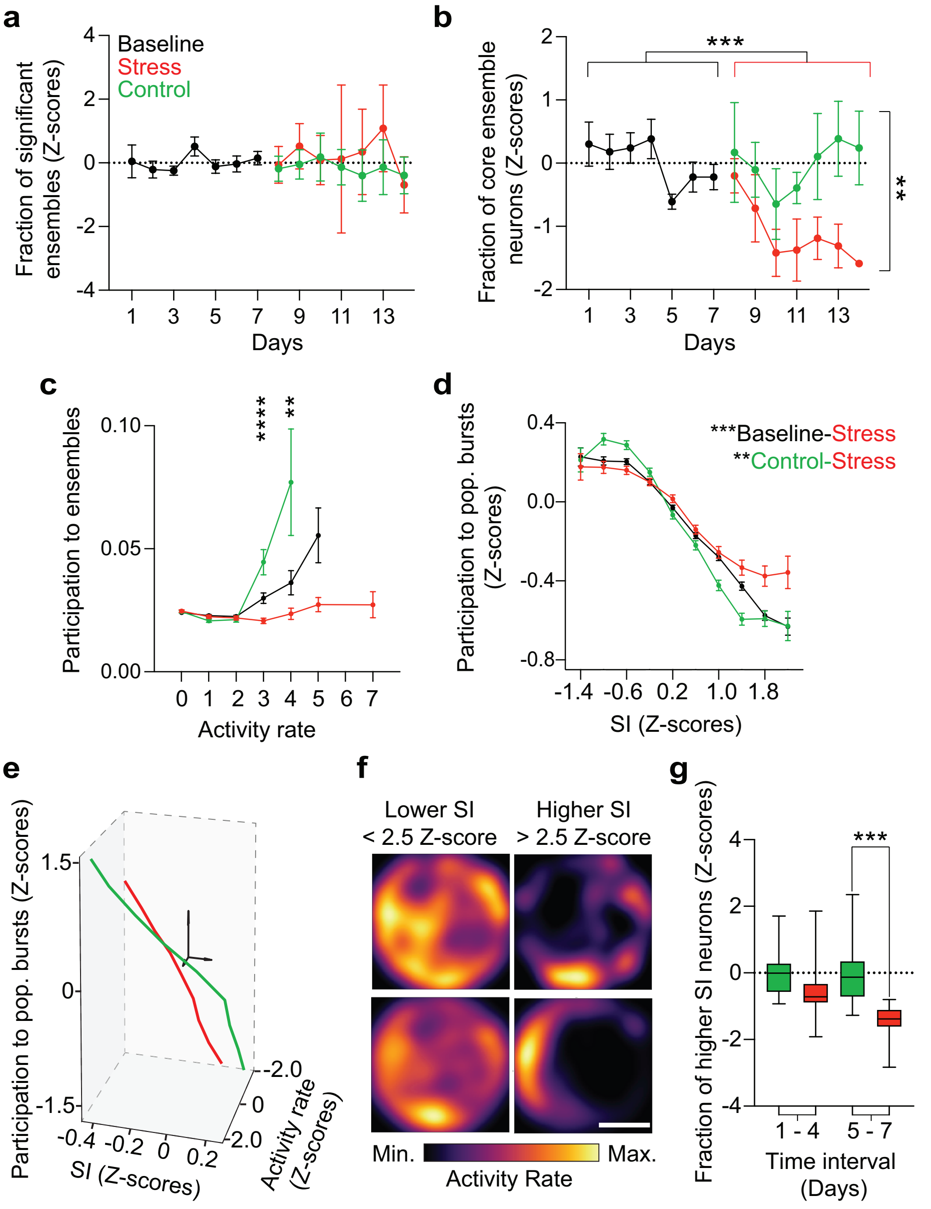

Supplementary Figure 5 | Exposure to repeated stress affects the temporal structure of activity and spatial coding in dCA1

(**a**) Stress exposure did not change the fraction of significant ensembles among all neurons ($p_{B\text{-}C} > 0.99$, $p_{B\text{-}S} > 0.99$, $p_{C\text{-}S} > 0.99$; $n_B = 84$, $n_C = 28$, $n_S = 45$).

(**b**) Stress exposure decreased the size of ensembles' cores for all neurons ($p_{B\text{-}C} > 0.99$, $p_{B\text{-}S} < 0.0001$, $p_{C\text{-}S} = 0.0017$; $n_B = 87$, $n_C = 24$, $n_S = 38$).

Circles: mean fraction of of significant ensembles per group (a) or core ensemble neurons per group (b). Error bars: s.e.m.

(**c**) Participation of high activity rate neurons in ensembles' cores increased with time, but stress exposure prevented this increase ($p_0 = 0.87$, $p_1 = 0.20$, $p_2 = 0.87$, $p_3 < 0.0001$; $p_4 = 0.038$; $n_0 = 14.970$, $n_1 = 9.423$, $n_2 =$

5.193, $n_3 = 2.326$, $n_4 = 718$, unpaired t-test, P values corrected for multiple comparisons with Holm-Šidák Method. Circles: mean ensemble participation index binned over activity rates. Error bars: s.e.m.

(**d**) Stress exposure affected the relationship between SI and the rate of participation in ensembles by decreasing the rate of participation in significant ensembles in neurons with lower SI and increasing the rate of participation in significant ensembles in neurons with higher SI ($p_{B-C} = 0.49$, $p_{B-S} = 0.0005$, $p_{C-S} < 0.0022$; $n_B = 9575$, $n_C = 25230$, $n_S = 1168$; Two-way ANOVA, p values adjusted after Tukey's corrections for multiple comparisons). Circles: mean ensemble participation index binned over activity rates. Error bars: s.e.m.

(**e**) Stress exposure shifted the state of the dCA1 PNs network in the three-dimensional space defined by activity, SI and participation in ensembles. The arrows in the center represent the axes originating at 0 with the arrows pointing in the positive direction.

(**f**) Example firing maps of two neurons with lower SI (left) and two neurons with higher SI (right) recorded in the same mouse during the same session. Scale bar, 20 cm.

(**g**) The difference between control and stress groups became significant after 5 days of repeated stress ($p_{8\text{-}11} = 0.075$, $p_{12\text{-}14} = 0.0007$; $n_{C\ 8\text{-}11} = 16$, $n_{S\ 8\text{-}11} = 20$, $n_{C\ 12\text{-}14} = 11$, $n_{S\ 12\text{-}14} = 9$). Box plots: medians and quartiles of the mean fractions of higher SI neuron distributions per mouse per session. Data in each time interval were normalized to the mean of the control group in that interval.

(**a ,b, g**) Kruskal-Wallis test, p values adjusted after Dunn's corrections for multiple comparisons.

(**b, f, g, h, i**), Circles: mean population bursts duration (b) modularity (e, f) or assortativity (h, i) per mouse. Error bars: s.e.m.

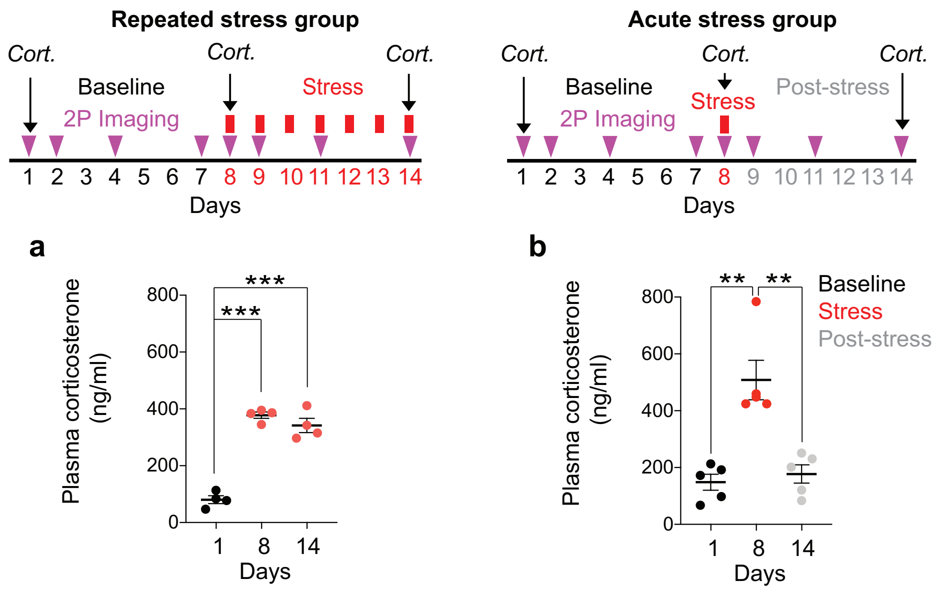

Supplementary Figure 6 | Repeated and acute stress exposures increase the blood corticosterone levels. **(a, b)** The blood levels of corticosterone in the blood of mice undergoing 2P optical imaging significantly increased in the Repeated ($p_{1\text{-}8}$ = 0.0001, $p_{1\text{-}14}$ = 0.0007, n = 12) and in the acute stress groups ($p_{1\text{-}8}$ = 0.007, $p_{8\text{-}14}$ = 0.007, n = 10). Repeated Measurements ANOVA, p values adjusted after Sidak's corrections for multiple comparisons).

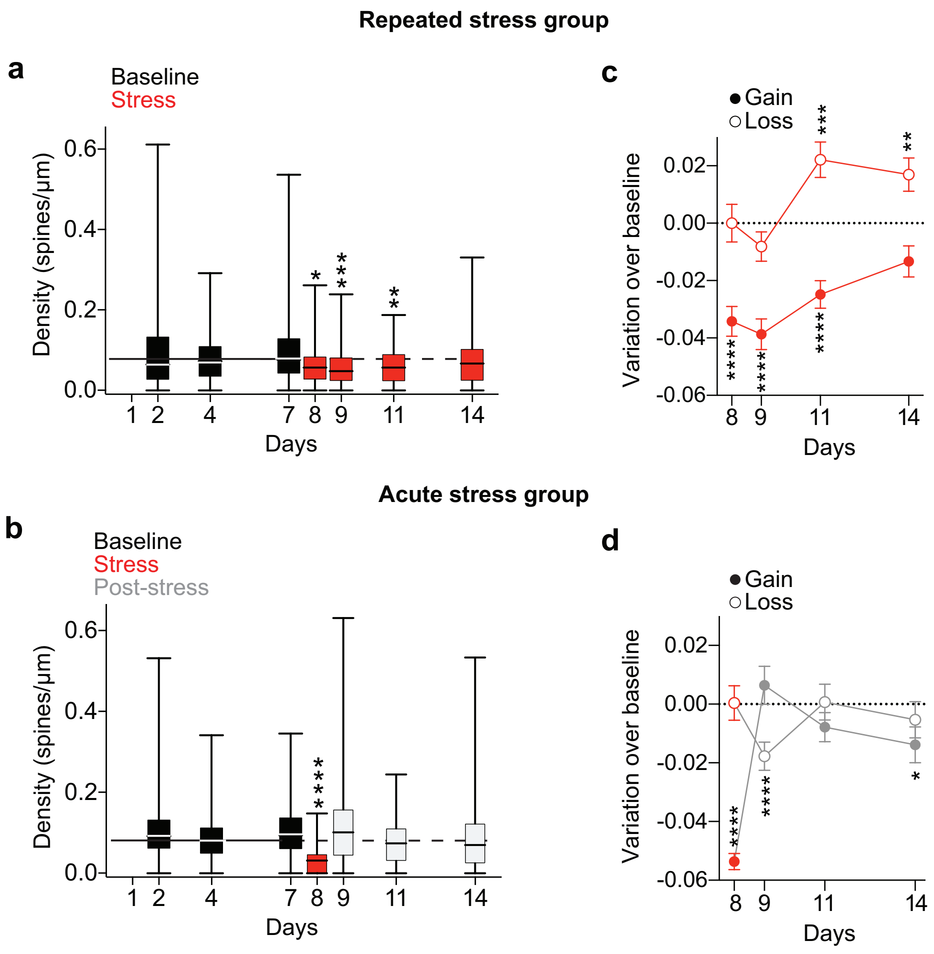

Supplementary Figure 7 | Stress affects spine dynamics proportionally to its duration

(**a**) The density of new spines decreased persistently during repeated stress exposure ($p_8 = 0.025$, $p_9 = 0.0002$, $p_{11} = 0.0018$, $p_{14} = 0.89$, $n_B = 248$, $n_{8, 9, 11, 14} = 114$).

(**b**) The density of new spines decreased immediately after single stress exposure but recovered after stress interruption ($p_8 < 0.0001$, $p_9 > 0.999$, $p_{11} = 0.057$, $p_{14} = 0.05$, $n_B = 352$, $n_{8, 9, 11, 14} = 88$).

Kruskal-Wallis tests against pooled baseline distribution, p values adjusted after Dunn's correction for multiple comparisons Box plots: medians and quartiles of spine densities distributions per dendrite. Black solid and dashed horizontal lines: mean density of spines during baseline.

(**c**) Repeated stress exposure initially decreased spine gain but later increased spine loss (gain, $p_{7\text{-}8} < 0.0001$, $p_{8\text{-}9} < 0.0001$, $p_{9\text{-}11} < 0.0001$, $p_{11\text{-}14} = 0.07$, n = 124; loss, $p_{7\text{-}8} > 0.99$, $p_{8\text{-}9} = 0.11$, $p_{9\text{-}11} = 0.0005$, $p_{11\text{-}14} = 0.0043$, n = 124).

(**d**) Acute stress decreased spine gain immediately and later increased spine loss (gain, $p_{7\text{-}8} < 0.0001$, $p_{8\text{-}9} = 0.33$, $p_{9\text{-}11} = 0.12$, $p_{11\text{-}14} = 0.026$, n = 124; loss, $p_{7\text{-}8} = 0.94$, $p_{8\text{-}9} = 0.0004$, $p_{9\text{-}11} = 0.91$, $p_{11\text{-}14} = 0.39$, n = 124)

One sample t-test against the value 0. Circles: mean variation per dendrites of spine gain (full) or loss (empty) over baseline of the corresponding time window.

Supplementary Table 1 | Parameter set used in CaImAn

Parameter name	Parameter value	Description
f_rate	45 Hz	movie frame rate
min_corr	0.8	minimum correlation of the peak
min_pnr	5	minimum peak to noise ratio
min_SNR	1.5	adaptive way to set threshold on the transient size
r_values_min	0.85	threshold on space consistency
decay_time	0.4	decay time of transients/indicator in Seconds
bord_px	15	pixels considered as border pixels from each side
dims	315, 315	dimensions of the movie to be processed
gSig	2	gaussian width of a 2D gaussian kernel, approximating a neuron
gSiz	9	average diameter of a neuron, in general 4*gSig+1
merge_thresh	0.7	threshold for merging
p	2	order of the autoregressive system
tsub	10	Down-sampling factor in time for initialization
ssub	1	Down-sampling factor in space for initialization
rf	40, 40	half size of the patch (final patch will be 100x100)
stride	20, 20	overlap among patches
gnb	-1	number of background components
nb_patch	8	number of background components per patch
method_deconvolution	'oasis'	Deconvolution method
ssub_B	2	additional down-sampling factor in space for background
ring_size_factor	1.5	radius of the ring
del_duplicates	True	whether to remove duplicates from initialization

Supplementary Table 2 | Number of neurons analyzed by WFHM imaging per mouse per day.

Control group

**Mouse ID:**	**1**	**2**	**3**	**4**
Day 1	564	227	39	
Day 2	629	392	135	439
Day 3	883	486	125	571
Day 4	907	668	114	564
Day 5	1072	679	227	520
Day 6	993	617	299	472
Day 7	1012	549	270	409
Day 8		651	253	341
Day 9	960	738	228	375
Day 10	981	941	208	323
Day 11	700	774	101	250
Day 12	664	795	81	317
Day 13	570	666	93	340
Day 14	625	658	114	310

Repeated Stress Group

**Mouse ID:**	**1**	**2**	**3**	**4**	**5**	**6**	**7**	**8**	**9**
Day 1	837	292	324	253	189			256	256
Day 2	600	316	286	180	171	286	302	242	355
Day 3	681	341	294	167	121	305	295	312	339
Day 4	725	417	305	179		380		221	287
Day 5	874	434	454	214	60	350	349	286	381
Day 6	951	407	371	246	88	317	331	305	328
Day 7	1028	426	412	262	19	407	280	248	322
Day 8	999	401	342	199		295	269	333	377
Day 9	1016	257	407	312	430	395	279	764	427
Day 10	1109	65	379	31	294	183	317	593	398
Day 11	1386	64	301	29	175				413
Day 12	1354	69	290	27	134				435
Day 13	1356	26	382	46	169				407
Day 14	1335	49	379	49	111				
